# Bypassing the Resistance Mechanisms of the Tumor Ecosystem by Targeting the Endoplasmic Reticulum Stress Pathway Using Ruthenium- and Osmium-Based Organometallic Compounds: An Exciting Long-Term Collaboration with Dr. Michel Pfeffer

**DOI:** 10.3390/molecules26175386

**Published:** 2021-09-04

**Authors:** Christian Gaiddon, Isabelle Gross, Xiangjun Meng, Marjorie Sidhoum, Georg Mellitzer, Benoit Romain, Jean-Batiste Delhorme, Aïna Venkatasamy, Alain C. Jung, Michel Pfeffer

**Affiliations:** 1 Université de Strasbourg-Inserm, UMR_S 1113 IRFAC, 67200 Strasbourg, France; gross@unistra.fr (I.G.); mellitzer@unistra.fr (G.M.); ben.romain@hotmail.fr (B.R.); jb.delhorme@hotmail.fr (J.-B.D.); aina.vnkt@gmail.com (A.V.); a.jung@icans.eu (J.A.C.); 2 Department of Gastro-Oncology, 7th Hospital, Shanghai 200137, China; xiangjunmeng@aliyun.com; 3 Domain Therapeutics, 67400 Illkirch, France; marjorie.sidhoum@gmail.com; 4 CNRS UMR 7177, Institute of Chemistry, 67000 Strasbourg, France; pfeffer@unistra.fr

**Keywords:** ruthenium, osmium, cisplatin, chemotherapy, TP53, ER stress pathway, immunotherapy, cancer, cell death, photodynamic therapy, resistance

## Abstract

Metal complexes have been used to treat cancer since the discovery of cisplatin and its interaction with DNA in the 1960’s. Facing the resistance mechanisms against platinum salts and their side effects, safer therapeutic approaches have been sought through other metals, including ruthenium. In the early 2000s, Michel Pfeffer and his collaborators started to investigate the biological activity of organo-ruthenium/osmium complexes, demonstrating their ability to interfere with the activity of purified redox enzymes. Then, they discovered that these organo-ruthenium/osmium complexes could act independently of DNA damage and bypass the requirement for the tumor suppressor gene *TP53* to induce the endoplasmic reticulum (ER) stress pathway, which is an original cell death pathway. They showed that other types of ruthenium complexes—as well complexes with other metals (osmium, iron, platinum)—can induce this pathway as well. They also demonstrated that ruthenium complexes accumulate in the ER after entering the cell using passive and active mechanisms. These particular physico-chemical properties of the organometallic complexes designed by Dr. Pfeffer contribute to their ability to reduce tumor growth and angiogenesis. Taken together, the pioneering work of Dr. Michel Pfeffer over his career provides us with a legacy that we have yet to fully embrace.

## 1. Introduction

Over the last few decades, the strategies to fight cancer have vastly improved due to the development of a wider diversity of therapeutic approaches, ranging from small molecules, specific antibodies, gene manipulations, and cellular therapies. Despite the amount of energy and massive investments committed in the search for improved anticancer therapies, this war is not over yet, as there are numerous cancers that remain dramatically lethal. For instance, gastric cancers display a dire clinical outcome, with a 5-year survival rate of between 10 and 30% in European countries (all stages taken together) and a median survival of 12 months [1]. In other words, it means that 50% of the patients die within a year after their diagnosis and it is not uncommon that patients die within 4 months after the diagnosis. This failure to efficiently treat these patients illustrates the complexity of cancer, especially the heterogeneity/diversity of cancers.

Indeed, despite the existence of common features shared by the different types of tumors affecting different organs, there are also key differences in terms of tissue structures and molecular alterations. For instance, cancers start with an activating mutation of an oncogene (e.g., *Ki-RAS)*, and an inhibiting alteration of a tumor suppressor gene (e.g., *TP53* (p53)). However, the identity of the key oncogene or tumor suppressor gene that drives the initial transformation process and/or maintains the transformed status (named “driver mutation”) varies between organs and even between the different tissues within a given organ. For instance, admittedly, inactivating mutations or alterations of p53 can be seen as a key primary event for the transformation of epithelial gastric cells, but it is not the same for glioblastoma or colon cancer, in which different tumor suppressor genes are primarily mutated (e.g., *APC* for colon cancer) [2]. In addition, the mutation or alteration affecting an individual gene may also differ between cancers. Typically, an inactivation of p53 can be caused by different point mutations, depending on the organ, but also by other means, such as an amplification of one of its negative regulators (e.g., *MDM2*), or the silencing of one of its activators (e.g., ***CDKN2A***). Additional tissue-specific factors can be altered and play a part in the tumorigenesis processes [3].

**Figure 1:**
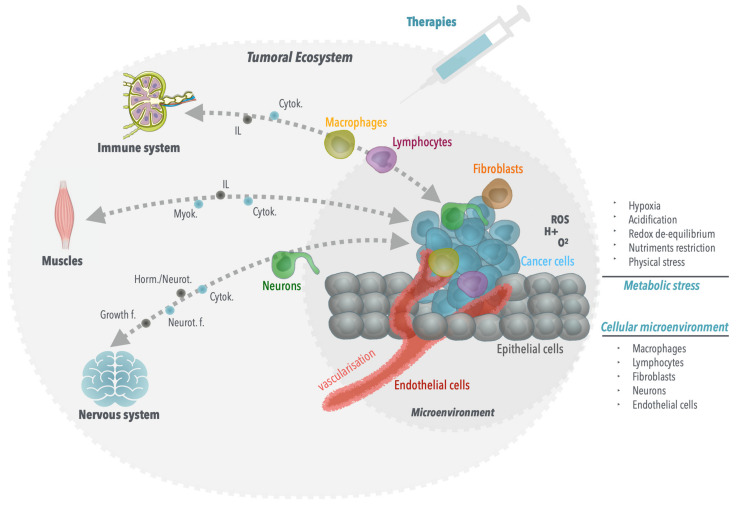
The tumor ecosystem. Cancer should be considered as a systemic disease as the tumor uses the entire organism for its own profit, akin to a parasite. First, upon growth, cancer cells can escape the immune system defense by inhibiting their own elimination by T-lymphocytes or M1 macrophages, while favoring the recruitment of regulatory lymphocytes and M2 macrophages, which produce tumor pro-proliferative cytokines and interleukins. These diffusible molecules produced by the tumor can negatively impact on the muscles, which in turn can also produce myokines, also impacting tumor growth. Following tumor growth, metabolic stresses, such as hypoxia, acidification, redox imbalance and nutrient deprivation drive a tumor response that induces a metabolic shift towards glycolysis and angiogenesis, through endothelial cell recruitment. In addition, in some cases, neurons or neuronal terminuses infiltrate the tumors and produce neurotrophic factors that favor tumor growth. Finally, the tumor surroundings and the multiple cellular intra-tumoral infiltrations produce a mechanical/physical stress on the tumor cells, which impacts on their behavior, resulting in their escape via metastatic processes.

Furthermore, we are starting to understand that cancer is not a localized disease, but rather a systemic and adaptive one that involves and affects the entire body through active cellular and molecular interactions, allowing tumor growth and causing resistances to therapy. This complex interaction starts within the tumor, in the immediate vicinity of the cancer cells where they interact with/use healthy cells for their benefit, such as capillary endothelial cells, fibroblasts, and immune cells (Figure 1). These interactions via direct contact with membrane proteins and diffusible molecules allow the cancer cells to adapt to their environment by changing their metabolism, recruiting blood vessels, escaping the immune response, allowing their survival through this plasticity. In addition, the tumor also interacts with distant tissues. For instance, gastric cancer development is significantly dependent upon innervation from the enteric nervous system [4]. Additionally, muscle atrophy is frequently associated with gastric cancer, correlating with a poorer prognosis [5,6,7,8]. More importantly, the development of the tumor depends on its ability to avoid/modify the immune system response. Hence, tumor growth and resistance to therapies involve multiple layers, starting with the molecular alterations of cancer cells, followed by interactions of the cancer cells with their immediate molecular and cellular environment (microenvironment) and then with distant organs (macroenvironment), all in all resulting in a complex and dynamic ecosystem capable of adaptation.

Hence, it is this complex ecosystem, as a whole, that responds and that contributes to define the efficiency of a given therapy. Indeed, beside the clonal selection of specific mutations in cancer cells, modifications of the metabolic and cellular microenvironment, as well as interactions with distant tissues, influence the response to therapy. Typically, treatment with cytotoxic drugs, such as oxaliplatin, favors the selection of gastric cancer cells harboring a p53 mutation or an overexpression of proteins involved in DNA repair mechanisms (e.g., ERCC1) [9], pumps (ATP7B, PGP) [10] or epigenetic regulators [11]. Treatments also affect the microenvironment by modifying the interaction with immune cells via the production of specific signals (e.g., DAMPs: Damage Associated Molecular Patterns). It also causes neuropathies [12] and muscle atrophy [5,6,7], impacting the ability of the patient to properly respond to therapy. These concerns are not limited to chemotherapies, but can be also applied to targeted therapies (e.g., anti-VEGFR or -EGFR antibodies therapies), which are also prone to similar resistance mechanisms [13]. In addition, targeted therapies bear the disadvantage of their elevated cost, representing an additional significant resistance towards their broad use in some countries, resulting in forms of social and racial inequity.

## 2. Organometallic Ruthenium Complexes as Regulators of Redox Enzyme Activity and Inducer of p53-Independent Cytotoxicity: Where the Story Began

Conventional cytotoxic drugs have two major disadvantages. Firstly, they lack selectivity towards the tumor as they often target DNA, a common macromolecule for all cells, leading to various side effects, including digestive problems, neuropathies, hair loss, irreversible sensorineural hearing loss, as well as cardiac, kidney and liver toxicities [14]. Secondly, about 50% of the cancers present mutations in the tumor suppressor gene *TP53* that plays an important role in the cellular response to DNA damage [15]. In this context, the organometallic compounds developed by Dr. Michel Pfeffer still are of particular interest, as our collective work demonstrated all along the years. 

Of course, as other previously investigated ruthenium complexes (e.g., Nami-A), the complexes developed by Dr. Michel Pfeffer bear all the advantages related to the ruthenium atom, including various charges, the possibility of six ligands, a ligand-Ru exchange rate compatible with biological applications, a wide range of reduction potentials and perhaps the possibility of reduced toxicity due to the proximity of Ru to Iron, which should be easily detoxified by the organism [16,17]. However, Michel’s complexes have additional properties due to the covalent bond between the Ru and the carbon atom, which may lead to an improved stability and modifies the range of reduction potentials.

In addition, Dr. Michel Pfeffer and his collaborator, Dr. A. D. Ryabov, discovered that these compounds were able to modify the activity of redox enzymes [18,19]. More precisely, the compounds reduced the activity of glucose oxidase but enhanced the activity of horseradish peroxidase via a catalytic mechanism. 

**Figure 2:**
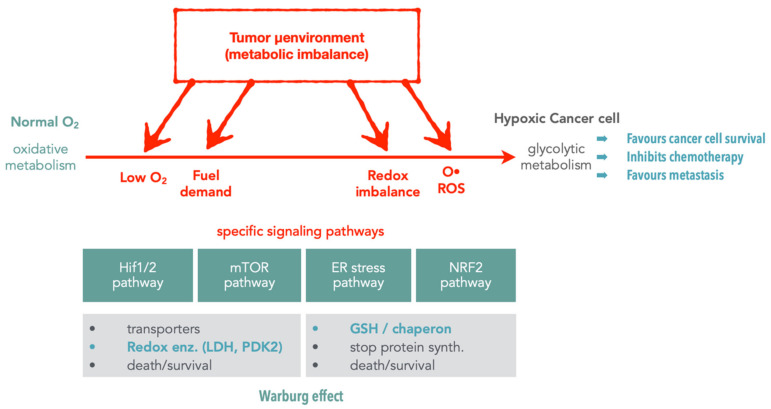
Metabolic/proteostasis pathways involved in tumor adaptation to their stressful environment. Upon metabolic imbalance (e.g., low oxygen content, elevated demand for fuel, redox imbalance, high production of ROS), cancer cells shift from an oxidative metabolism toward a glycolytic one induced by different pathways that detect the metabolic imbalances. These pathways include the HIF1/2, mTOR, ER stress and NRF2 mechanisms that contribute to the modification of the expression of transporters (ex. glucose transporters), redox enzymes (ex. LDH, PDK2), protein chaperones, and antioxidant enzymes (ex. GSH). This coordinated response allows cancer cells to survive.

These early observations were particularly interesting, as redox enzymes play a crucial role in cancer cell survival [20,21]. Indeed, upon tumor growth, cancer cells progressively develop further away from blood vessels, leading to reduced oxygen availability. Hence, in response to this hypoxia (i.e., lack of oxygen), cancer cells develop a hypoxic response via the hypoxia response transcription factor HIF1A (Figure 2). This transcription factor is a key mechanism that induces the expression of VEGF, a growth factor stimulating vascularization and the expression of glucose transporters (i.e., GLUT1) and redox enzymes (e.g., LDH, lactate dehydrogenase), which allow the cells to shift from the oxidative metabolism towards the less-efficient glycolytic metabolism, which does not require as much oxygen. This process is called the Warburg effect. Consequently, targeting redox enzymes involved in the oxidative-to-glycolytic metabolism shift constitutes a promising strategy to selectively target cancer cells without affecting the surrounding healthy tissues, thereby also potentially bypassing classical mechanisms of resistance against DNA damage.

**Figure 3:**
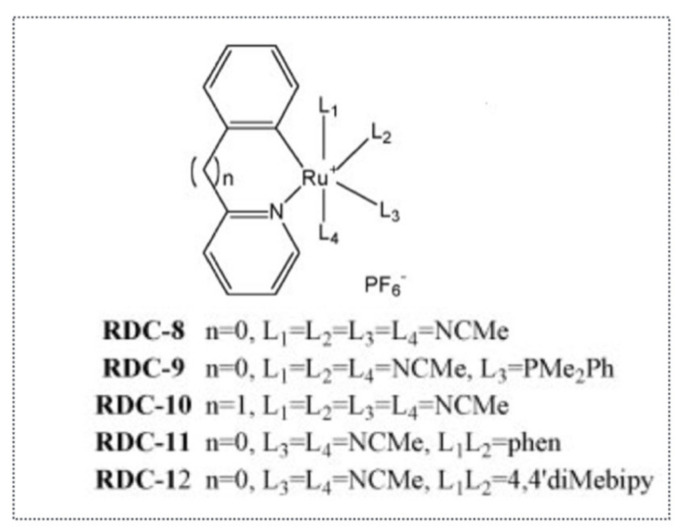
Examples of ruthenium organometallic complexes that induce the ER stress pathway. First generation RDC synthesized by the Dr. M. Pfeffer’s team and tested for their biological properties.

Hence, we first investigated the cytotoxic activity of eight complexes with Ru-C bonds on seven cancer cell lines of different origins (e.g., glioblastoma, colon cancer, lymphoblastoma). These complexes were named “RDC” for Ruthenium-Derived Compounds (Figure 3). Three of those complexes had a strong cytotoxicity, similar to or higher than cisplatin [22]. We performed a more detailed investigation of the molecular mode of action involved in the cytotoxicity and determined that both in the glioblastoma cells (A172) and the colon cancer cells (HCT116) several complexes induce apoptosis via cleavage of caspase 3. We also showed that one of the complexes, RDC9, increases the protein levels of the tumor suppressor gene *TP53* and of one of its homologues, TAp73. However, we demonstrated that the pro-death activity of RDC9 was p53-independent, using the expression of dominant inhibitory forms of p53 (DDp53 or ∆Np73) or deletion of the *TP53* gene.

***This represented the first evidence that a ruthenium complex with a Ru-C bond was able to kill cancer cells, independently of the activity of p53. This was a critical finding, as TP53 is mutated and inactivated in more than 50% of tumors.***

## 3. Organometallic Carbon-Ruthenium Complexes as Inducers of the Reticulum Endoplasmic Stress to Target Cancer Cells

Unfortunately, RDC9 showed limited stability. In contrast, RDC11 had a relatively simple synthesis and purification process associated with good stability, and showed an IC_50_ below 5 µM in all the tested cell lines. Based on these chemical properties, we analyzed in further detail the biological properties of RDC11. Our study had three important objectives: (1) establish whether RDC11 had anticancer properties in vivo using animal models; (2) assess its toxicity on healthy cells and healthy tissues; (3) identify the molecular mechanisms used by RDC11 for its cytotoxicity. Using three different animal models, a melanoma syngeneic model (B16F10), a glioblastoma xenograft model (A172) and an ovarian cancer xenograft model (A2780), we demonstrated that RDC11 was able to reduce tumor growth by more than 50%, with an efficacy similar to or higher than cisplatin [23]. Interestingly, in vitro, RDC11 was less cytotoxic to healthy glial cells maintained in ex vivo cultures compared to glioblastoma cells. In addition, blood analysis as well as electromyography showed that RDC11 induced significantly fewer side effects in the liver, kidneys and peripheric nervous system compared to cisplatin following chronic injection in mice.

The analysis of the molecular mechanisms underlying RDC11 action indicated that RDC11 was less able to interact with DNA or induce DNA damage compared to cisplatin, as indicated by the phosphorylation of H2AX or in vitro direct DNA interaction. Detailed analyses showed that, in contrast to cisplatin, RDC11 did not interact covalently with DNA but rather intercalates [24]. This led to a reduced activation of p53, despite the induction of growth arrest and cell death. Hence, we searched for alternative molecular mechanisms that might be responsible for cell death induction by RDC11. As Dr. Pfeffer and Dr. A. D. Ryabov found that ruthenium complexes were potential regulators of redox enzymes, we hypothesized that RDC11 may also interfere with the activity of redox enzymes and that it might in turn impact on the redox status of cancer cells. All this would result in protein oxidation that would cause misfolding and an induction of the Unfolded Protein Response (UPR), also called the Endoplasmic Reticulum Stress (ER stress) pathway.

**Figure 4:**
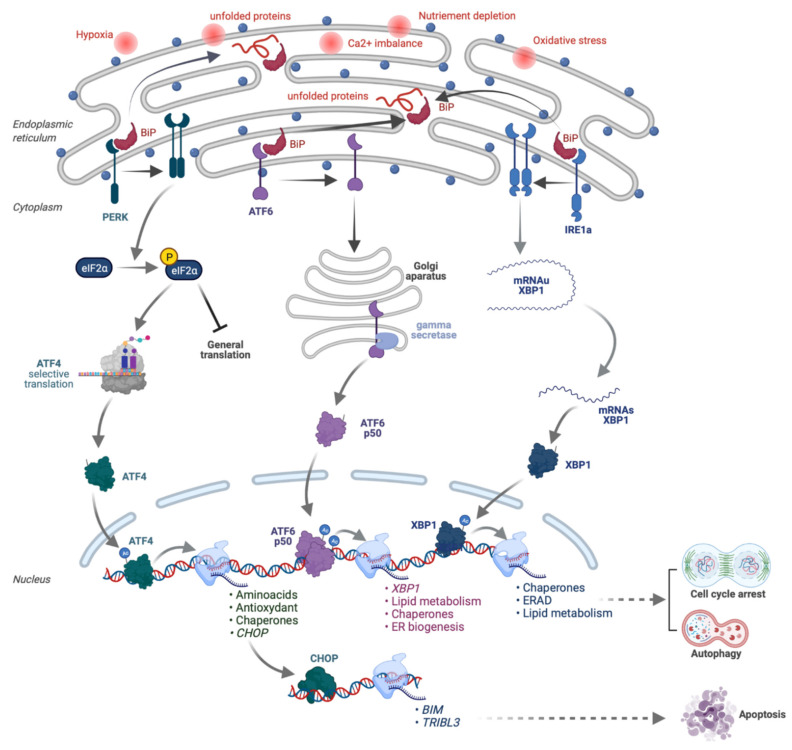
Reticulum endoplasmic stress response pathway. Designed with Biorender.

The function of the ER stress pathway is to maintain protein homeostasis in cells and to settle on their survival. It is activated by the accumulation of **misfolded** proteins, caused by oxidation or deficiency of nutrients (e.g., O_2_, amino acids), and it organizes an adapted response: (1) **cell survival** by reducing overall protein translation, while ensuring the expression of “chaperones” that “repair” misfolded proteins and (2) **elimination** of the over-damaged cell by inducing cell death, either by **apoptosis** or **autophagy** [25,26] (Figure 4).

The **ER stress pathway** includes **three molecular cascades** controlled by “**sensor**” **proteins** inserted in the membrane of the ER: (i) **PERK,** initiating an Eif2a-ATF4 cascade; (ii) **ATF6,** whose cleavage generates an ATF6c fragment; (iii) **IRE1a**, which induces the alternative splicing of XBP1 into **XBP1s**. The balance between the transcription factors **ATF4**, **ATF6c** and **XBP1s** determines the cell fate via target genes involved in the synthesis of amino acids, antioxidants, chaperones, or on the contrary the expression of pro-cell death genes (e.g., ***CHOP***, ***BIM1***, ***PUMA***). The ER Stress pathway modulates the response to cancer treatments. The ER stress pathway has an ambivalent role in cancers. Mutations in some of its constituents promote tumorigenesis, suggesting that they have **tumor suppressor functions** [27]. In contrast, **activation of the ER stress pathway** is also described as **a resistance mechanism against chemotherapies** (e.g., overexpression of pumps). However, specific chemotherapies (e.g., oxaliplatin) may induce, through the ER stress pathway, **immunogenic cell death** characterized by **danger molecules** (**DAMPS**; e.g., **CALR** membrane relocation) that **activate the immune system**, potentially improving the response to **immunotherapies** [26]. Based on these complex effects on tumor growth, a **large collection of inducers and inhibitors of the ER stress pathway** have been developed to target cancer. The ER stress pathway is also actively involved in the immune response and, in particular, in the expression of various cytokines. For example, the transcription factor **CHOP**, a major effector of the ER stress pathway, stimulates the **IL6/STAT3** pathway [28,29]. **IRE1a** modifies the activity of the major inflammation regulators **IKK** and **NF-KB** via the recruitment of **TRAF2**. **PERK** interacts with **JAK1** to induce **STAT3** and the expression of cytokines, such as **IL6**. In fact, the major role of the **ER stress pathway in cellular responses** has already been demonstrated for some viruses, such as for the Newcastle disease virus [30,31].

Thus, we analyzed the expression of key biomarkers of the ER stress pathway. We found that several biomarkers of this pathway were induced by RDC11, such as Bip, XBP1, CHOP and PDI (Protein Disulfide Isomerase) [23]. Importantly, the induction was similar to what was observed when cells were treated with tunicamycin, a well-described inducer of ER stress that inhibits protein glycosylation leading to protein misfolding. Hence, the intensity of the induction suggested that RDC11 was a strong inducer of at least some markers of ER stress and that it might be physiologically relevant (Figure 4).

Very importantly, in our hands, cisplatin was not able to induce these markers of ER stress. Hence, RDC11 represented the first example of a metal-based compound able to induce the ER stress pathway, which represented a different pathway than the one induced by platinum-based drugs.

Importantly, we observed that RDC11 was able to strongly induce CHOP, a transcription factor of the CCAAT/enhancer binding protein (C/EBPs) family that mediates apoptosis in response to ER stress [23]. We observed that the mRNA and the protein levels of CHOP were induced, which led to an increased expression of its target genes *TRIB3* and *CHAC1* via its direct binding to the *TRIB3* promoter. These results led to the question as to whether the induction of the ER stress pathway, in particular of CHOP, was just a bystander or an active contributor to RDC11 cytotoxicity. To address this, we performed a functional loss of function experiment using siRNA against CHOP and found that the silencing of CHOP reduced the cytotoxicity of RDC11. This clearly showed that the ER stress pathway, in particular CHOP, was a key mediator of the RDC11 biological activity.

***These findings were quite an important step for our organoruthenium compounds, because they were the first demonstration that a ruthenium complex could exert its anticancer activity with less toxicity than platinum compounds, via an original signaling pathway, the ER stress pathway, and that this complex could bypass the requirement for DNA damage and the activity of the tumor suppressor gene p53 (that is mutated in 50% of cancers) to induce cytotoxicity in cancer cells.***

Based on these exciting data, Dr. Michel Pfeffer, Dr. Marjorie Sidhoum, a postdoc in our lab, and Dr. Christian Gaiddon started a company called Almetis aiming at the development of ruthenium complexes to cure cancer, subsequently winning a national competition for innovation and received a prize of more than 300,000 Euros. Unfortunately, the financial crisis reduced the Almetis’s chances to raise private funding and ability to take part in the competition for targeted-therapy engouement. In addition, Almetis’s efforts were also limited by the intrinsic ability of RDC11 to significantly reduce tumor growth in vivo. Indeed, RDC11 reduces tumor growth by at least as much as cisplatin, but not significantly better. Based on this experience, we aimed to further develop the chemistry of ruthenium complexes and endeavored to find complexes with increased activity.

## 4. Ruthenium Complex Activity Depends on Reduction Potential and Lipophilicity

**Figure 5:**
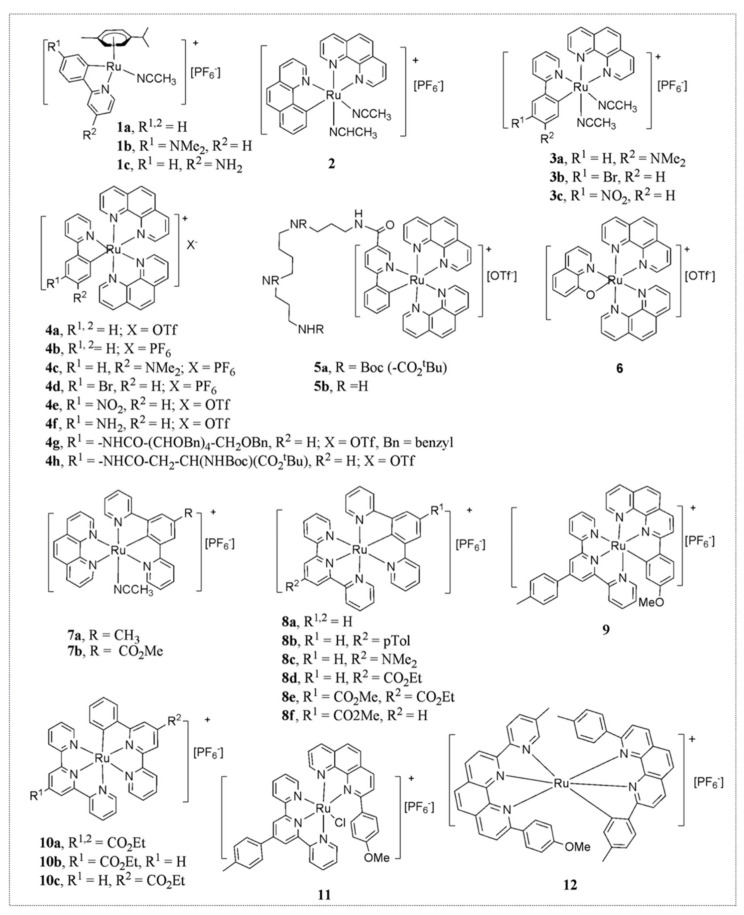
Examples of ruthenium organometallic complexes that induce the ER stress pathway. Second generation of RDC synthesized by the Dr. Pfeffer and tested for their biological properties.

Hence, Michel’s lab developed a second-generation library of organo-ruthenium complexes that contained about 30 novel chemicals with different reduction potentials and various degrees of lipophilicity. Several of these complexes displayed higher cytotoxicity than RDC11 with IC_50_ within the nanomolar range [32] (Figure 5).

A detailed structure-activity relationship analysis indicated that the cytotoxicity of the complexes was dependent both on the lipophilicity and reduction potential. The fact that the activity was lipophilicity-dependent is not surprising as it likely impacts the ability of the complexes to pass through the biological membranes via passive diffusion, especially the plasma membrane, and accumulate in higher quantities within the cell. For instance, a lipophilicity with a *log p* above 2 gave the highest cytotoxicity. However, the fact that the reduction potential may play a role was rather interesting, especially since we observed that complexes having reduction potentials between 0.4 and 0.6 V were particularly cytotoxic [32]. The biological significance of this redox window remains elusive. Since Dr. Pfeffer and Dr. A. D. Ryabov previously demonstrated that ruthenium complexes may interfere with redox enzymes, it is tempting to speculate that those redox enzymes exerting their activity within this redox range might specifically be affected by ruthenium complexes. However, it is also possible that ruthenium complexes drive chemical reactions on their own, and not specifically by reacting with chemical intermediates derived from various redox reactions. Addressing these two possibilities remains a challenge.

**Figure 6:**
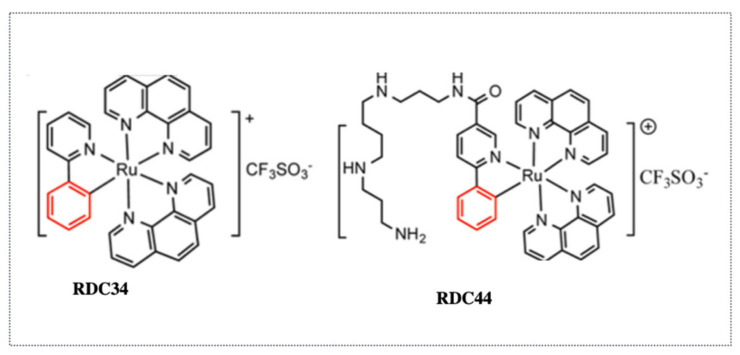
Ruthenium organometallic complexes RDC34 and RDC44 with fluorescent properties used for intracellular localization and cell entry.

Based on the encouraging in vitro results provided by this second generation of ruthenium complexes active at the nanomolar range, we investigated their in vivo activity and their mode of action. One of the compounds, RDC34, was particularly cytotoxic, with an IC_50_ below 1 µM in almost all tested cell lines, including in the NCI cancer cell line panel [33] (Figure 6). RDC34 is a complex in which the two acetonitrile ligands of RDC11 are substituted by a second phenanthroline (Figure 1). RDC34 is more potent at inducing apoptosis compared to RDC11 without an increased interaction with DNA, highlighting again that the main mode of action of RDC11 and RDC34 is not to target DNA. However, RDC34 also induces ER stress markers, such as CHOP. This leads to apoptosis through the intrinsic apoptosis pathway via mitochondria depolarization and induction of *NOXA*, and via the extrinsic apoptosis pathway, through an activation of *FAS* and caspase 8 cleavage. In addition, we showed that RDC34 induces the production of Reactive Oxygen Species (ROS). Although the in vitro results were highly encouraging, the in vivo data were a bit disappointing. Namely, the toxicity of RDC34 was higher compared to RDC11, reducing the maximal tolerated dose that could be injected into animals. In the end, RDC34 did not display higher anticancer efficacy against tumor growth in syngeneic 3LL (lung cancer) or xenograft A2780 (ovarian cancer) models.

## 5. Ruthenium Complexes Use Passive and Active Transport Mechanisms to Enter Cancer Cells and Localize in the Endoplasmic Reticulum and the Mitochondria

A particularly interesting feature of RDC34 was its intrinsic fluorescent property at about 720 nm. We exploited this property to investigate how ruthenium complexes enter the cells and to visualize their subcellular localization. After 1 h, RDC34 is colocalized with endoplasmic reticulum markers and mitochondrial markers, such as *SATB2* and *HSP60* [24]. In addition, this localization coincides with the induction of ER stress markers, as well as mitochondrial stress markers. Thus, we can hypothesize that part of the ability of ruthenium organometallic complexes to induce markers of the ER stress pathway is due to their tendency to accumulate within the endoplasmic reticulum.

Several previous studies correlated the toxicity of ruthenium complexes with their lipophilicity, suggesting that these compounds were entering the cells via passive transport through the plasma membrane. An alternative hypothesis is that the ruthenium complexes may use the iron transport mechanisms as Ru belongs to the same chemical family as Fe. However, up to then, there was no solid experimental evidence in cells or in vivo supporting this latter possibility. Using the fluorescent properties of RDC34, we observed that the rate of entry of ruthenium complexes within non-cancerous cells was lower compared to the rate measured within cancer cells. This observation provided a first explanation about why some ruthenium complexes may be less toxic to healthy tissues. It also suggests that in addition to passive diffusion, additional mechanisms of entry might be involved. Interestingly, we observed that at a lower concentration of RDC34 (1 µM), the active transport using ATP accounted for up to 50% of the entry. Hence, we explored the possibility that RDC34 was using the iron transport mechanism. We observed that RDC34 treatment increases the levels of Ferritin, which stores iron in the cells, while reducing Transferrin expression, which allows the entry of iron into cells [34]. This suggested that the cells perceived RDC34 as if it were iron, which would indeed explain a preferential accumulation in cancer cells in comparison to healthy cells. More precisely, it is well known that due to their special metabolism (i.e., the Warburg effect), the cancer cells present a particularly high use of metabolites, including iron. Interestingly, by chelating iron using deferoxamine, we increased the entry of RDC34. This set of data suggested that RDC34 was at least partially using the iron transport mechanisms to enter cells (Figure 6).

In addition, we also observed that other transporters were regulated upon RDC34 treatment. In particular, SLC7A5, an aminoacidic transporter, was induced. Its inhibition using a D-phenylalanine led to a reduced entry of RDC34. This indicated that SLC7A5 was also used by RDC34 to enter cancer cells. It represented the first evidence that an amino acid transporter could be a mechanism of transport for ruthenium complexes. 

***Hence, ruthenium complexes are using multiple mechanisms, both passive and active ones, to enter cells. This has a profound importance when considering the design of anticancer complexes, as it highlights the fact that the complexes should not be overlooked as simple lipophilic compounds that enter the cells via passive mechanisms. Actually, the fact that our complexes may use different active transport pathways opens up avenues to design complexes that display more selectivity towards cancer cells.***

## 6. Activation of the ER Stress Pathway Is a Common Feature of Several Types of Ruthenium Complexes

**Figure 7:**
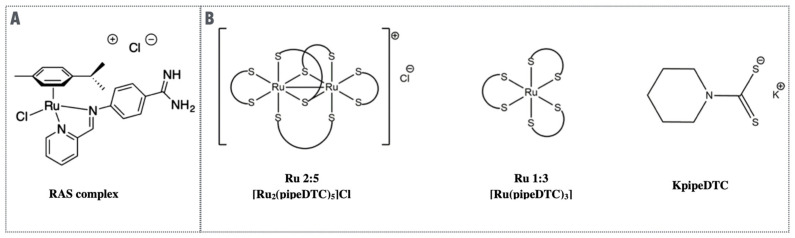
(**A**). Ruthenium complexes with piano-stool structure. (**B**). Ruthenium complexes with bond to sulfur. Ru 2:5 and Ru 1:3 were synthesized using the ligand K pipeDTC represented on the right. All these complexes can induce the ER stress pathway.

Our discovery that the ER stress pathway was induced by ruthenium complexes with a Ru-C bond raised the question of whether these types of complexes were the only ones that possess such ability. Hence, while analyzing the mode of action of the ruthenium complexes RAS1T and RAS1H, developed through a collaboration with Professor Wee Han Ang (NUS, Singapore), we investigated whether they could also induce the ER stress pathway. RAS1T and RAS1H are complexes with a piano stool structure that is very different from RDC11 or RDC34 and that have a η6 Ru-C binding. These complexes are Ru (II)-arene Schiff-Base (RAS) structures (Figure 7). The complexes were initially identified via a combinatory chemistry synthesis approach, coupled with a high-throughput screening process for detecting highly cytotoxic molecules [35]. Our studies revealed that both complexes were able to induce several markers of the ER stress pathway, such as XBP1s and CHOP. In addition, these compounds induced ROS and a ROS response via NRF2 induction [36]. NRF2 is a transcription factor that favors the expression of genes that can counteract the deleterious impact of ROS. Interestingly, we showed that only one of the complexes, RAS1T, required the production of ROS to induce cell death and the expression of the ER stress markers. Furthermore, we demonstrated that the modification of the iminoquinoline ligand tunes its π-acidity. It also influences reactive oxygen species (ROS) induction, switching from a ROS-mediated ER stress pathway activation to one that is not mediated by ROS induction [37]. Hence, this study showed that ruthenium complexes can induce the ER stress pathway via at least two different mechanisms, one that depends on the production of ROS and a ROS-independent one.

## 7. Do Other Metal-Based Complexes Induce ER Stress?

**Figure 8:**
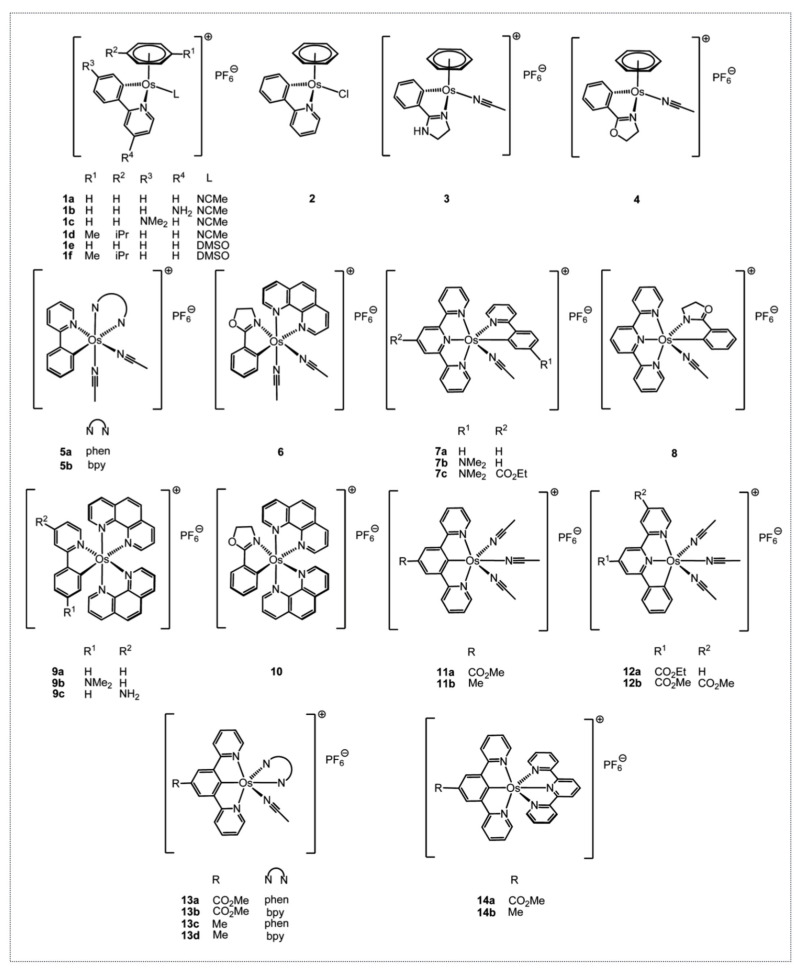
Osmium organometallic complexes designed and synthesized by Dr. Michel Pfeffer.

Based on the interesting anticancer properties of the organo-ruthenium complexes Dr. Michel Pfeffer synthesized, he wanted to determine if other metals bearing similar physico-chemical properties would also potentially be promising cytotoxic compounds. Osmium was chosen and a first series of novel and previously described complexes were synthesized, as a result of which we showed that they displayed a cytotoxic activity driven by the level of lipophilicity and the level of reduction potential [38,39]. Several complexes had a cytotoxicity with an IC_50_ below 1 µM (Figure 8).

To further assess the role of the metal in the biological activity of the complexes, the Os complexes were tested comparatively to their Ru counterparts in several cancer cell lines and their mode of action was analyzed [40]. ODC2 and ODC3, the osmium counterparts of RDC11 and RDC34, respectively, were chosen. In addition to the in vitro cytotoxicity, both complexes reduced in vivo tumor growth by about 40% in a syngeneic model of melanoma. Interestingly, the osmium counterparts of RDC11 and RDC34, ODC2 (5a on Figure 8) and ODC3 (9a on Figure 8), respectively, exhibited a higher cytotoxicity on the 60 cancer cell lines of the NCI, regardless of the cancer type. The cytotoxicity was associated with reduced cell growth and increased cell death. At the molecular level, p53 protein levels were not induced and p53 activity is not required for the cytotoxicity of the organo-osmium complexes, in contrast to what we previously observed for ruthenium complexes. Similarly, two osmium complexes were able to induce the pro-apoptotic marker of the ER stress pathway, CHOP. Interestingly, an osmium complex with a similar ligand to ODC2 and ODC3 but with no Os-C bond was not able to induce CHOP but induced p53 [41]. 

**Figure 9:**
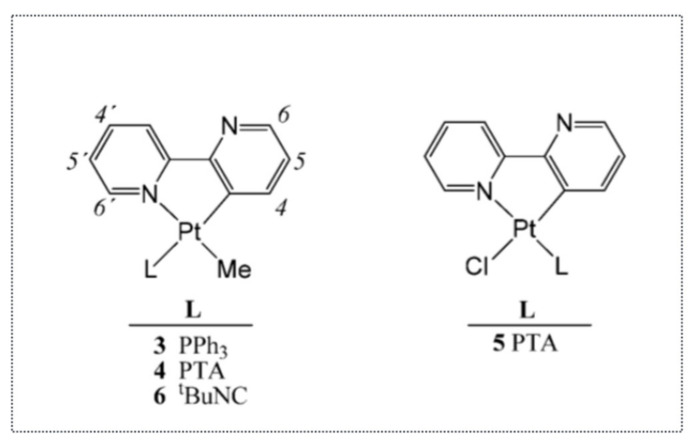
Platinum organometallic complexes inducing the ER stress pathway.

Thus, these studies clearly indicated that organo-osmium complexes were able to induce cancer cell death via the ER stress pathway, independently from the tumor suppressor gene p53. It also indicated that this property was somehow more often found in complexes with a metal–carbon bond. In addition to organo-osmium or organo-ruthenium complexes, we also showed, in collaboration with Dr. Christian Hartinger (U. Auckland, New Zealand), that organo-platinum complexes can also induce the expression of ATF4, an early marker of the ER stress [42] (Figure 9). More precisely, rollover cyclometalated Pt compounds based on 2,2′-bipyridine are potent antitumor agents both in vitro and in vivo. Variation of the coligands on the Pt (2,2′–bipyridine) backbone resulted in the establishment of structure—activity relationships. The less stable compounds caused higher reactivity with biomolecules and were shown to induce DNA damage and p53. In contrast, the presence of bulky 1,3,5-Triaza-7-phosphaadamantane (PTA) and PPh_3_ ligands caused lower reactivity and increased antineoplastic activity. Such compounds were devoid of DNA-damaging activity and induced ATF4, a component of the ER stress pathway.

However, not all metals are equivalent. For instance, we recently showed that ruthenium and gold complexes with similar ligands did not trigger the same molecular mechanisms, which is well illustrated by [AuCl_2_(pipeDTC)] and β-[Ru_2_(pipeDTC)_5_]Cl [43] (Figure 7B). The first compound was able to induce the DNA damage response factor p53 and the autophagy protein p62. In contrast, the second compound induced the ATF4 protein (a marker of the ER stress pathway), but repressed p62 expression. This study highlights that the biological activity of different complexes presenting the same organic ligand depends on the electronic and structural properties of the metal, which are able to fine tune its biological properties, providing us with precious information that can help designing of more selective anticancer drugs. 

***Thus, the ER stress pathway seems to be a pathway particularly sensitive to organometallic complexes***.

## 8. Why Do Ruthenium Complexes Induce the ER Stress Pathway? Are Redox Enzymes Targeted by Ruthenium Complexes?

**Figure 10:**
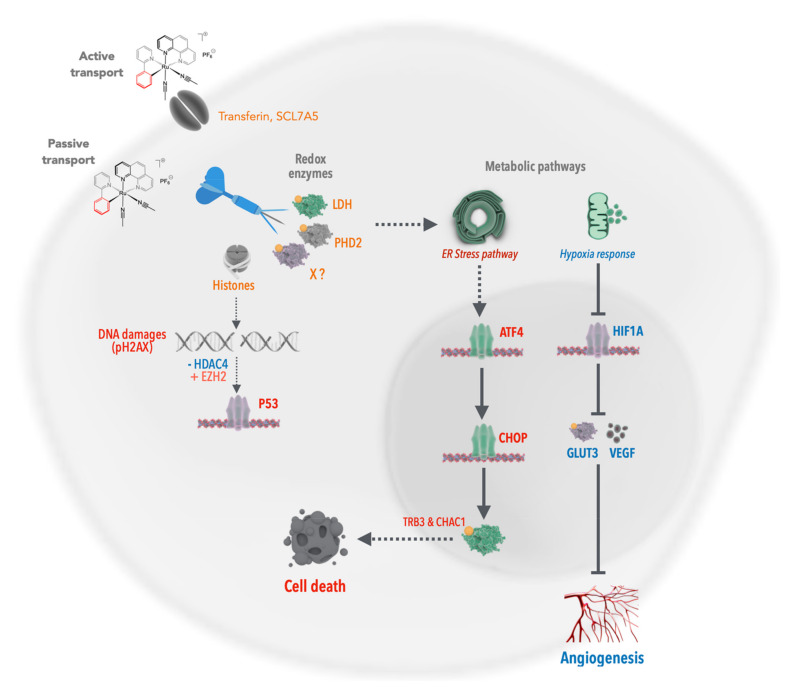
Up-to-date knowledge on the mode of action of ruthenium and osmium complexes.

One of the critical questions is how do organo-ruthenium complexes induce ER stress? Our results indicate that one way is through the induction of radical oxidizing species (ROS), in the case of RAS1T [37]. This finding is in agreement with previous studies showing that ROS can induce ER stress. Indeed, protein oxidation can cause misfolding, leading to protein aggregates, which cause ER stress. However, RAS1H and other Ru complexes do not seem to require ROS production to induce cell death or ER stress. Our initial hypothesis was that Ru complexes may directly impact on the activity of redox enzymes that are essential for cancer cell survival. Therefore, we tested this hypothesis on the enzyme LDH (Figure 10). 

Indeed, a distinctive feature of cancer cells is their elevated production of lactate caused by high glucose import and the switch to glycolic metabolism [21]. This Warburg effect is of great importance. The metabolic changes in the tumor microenvironment (i.e., hypoxia and low pH) play crucial roles by regulating specific signaling pathways including HIF1 (hypoxia inducible factor 1) and the unfolded protein response pathway (Figure 2). These pathways are essential to ensure the survival of cancer cells through the control of (i) the activity and the expression of redox enzymes (oxidoreductases) involved in the production of ATP/energy and (ii) the biosynthesis of essential elements (amino acids, nucleic bases) for biomacromolecules. Lactate dehydrogenase (LDH), which catalyzes the production of lactate in the final step of the glycolytic pathway [44], is one of these key enzymes [45]. The importance of LDH is illustrated by the elevated expression of the *LDHA* gene in multiple tumors. Furthermore, the inhibition of *LDHA* expression reduces tumor aggressiveness and resistance to chemotherapy [46]. Hence, LDH is emerging as an interesting anticancer target [47]. To further decipher the anticancer activity of RDC, we decided in collaboration with Dr. R. Le Lagadec (UNAM, Mexico) to analyze their impact on the activity of LDH both in vitro using purified enzymes and in cancer cells. Two structurally very similar compounds, having only a noticeable difference in their reduction potential, were used to further understand the physicochemical determinant essential for their biological activity. For instance, a polypyridine ruthenium(II) complex [Ru(bpy)_3_]^2+^ and its structurally related cyclometalated 2-phenylpyridinato counterpart [Ru(phpy)(bpy)_2_]^+^ (bpy = 2,2’-bipyridine, phpyH = 2-phenylpyridine) were used. As previously observed, the cyclometalated complex had a higher cytotoxic effect on gastric cancer cells. Using purified enzymes and measurements of LDH activity in cancer cells, we showed that both complexes were able to reduce the activity of LDH. Interestingly, only the cyclometalated complex behaved exclusively as a non-competitive inhibitor of LDH_rm_, strongly suggesting that it does not interact with LDH in the vicinities of either the lactate/pyruvate or NAD^+^/NADH binding sites but by another mechanism. One hypothesis is that it may involve the redox property of the complex. Hence, ruthenium complexes can achieve the inhibition of the activity of redox enzymes, such as LDH, through a direct interaction structurally tuned by a Ru-C bond (Figure 10).

If ruthenium complexes can inhibit the activity of redox enzymes, the reverse is also true. Dr. A. D. Ryabov and Dr. R. Le Lagadec had previously showed that depending on the oxidation state, the ruthenium complexes may have opposite effects on the enzyme, glucose oxidase depending [48,49]. We recently extended that observation showing that RDC11 can favor the activity of the iron redox enzyme PHD2 (prolyl hydroxylase domain-containing protein 2) and that this has a profound impact on the biological activity of RDC11 as an anticancer agent. We identified PHD2 as a possible target through a transcriptomic analysis of cancer cells treated with RDC11, which was aimed at the identification of deregulated signaling. One of the pathways that was inhibited by RDC11 was the HIF1A-dependent pathway, as illustrated by several target genes of HIF1A that were downregulated by RDC11 [50]. As indicated above, HIF1A is a crucial transcription factor induced in cancer cells in response to hypoxia. Its protein level is tightly controlled by the PHD enzymes, in particular PHD2, that cause its hydroxylation in the presence of sufficient O_2_, leading to HIF1A degradation by the proteasome pathway. PHD enzymes are redox enzymes that contain an iron atom essential for their function. As the protein level of HIF1A was reduced in cancer cells treated with RDC11, we hypothesized that RDC11 might modify the activity of PHD2. First, we checked that the protein level of PHD2 was not affected. Then, we measured the activity of PHD2 and observed that RDC11 stimulated its activity, which resulted in an increase in hydroxylation of HIF1A followed by its degradation. The exact mechanisms of action used by RDC11 to induce PHD2 activity remain unclear. However, previous studies conducted by Dr. A. D. Ryabov showed that in the case of the horseradish peroxidase, ruthenium complexes may contribute to the reactivation of the enzyme [51].

Hence, ruthenium complexes can inhibit or increase the activity of various redox enzymes that play a critical role in cancer cell survival, enabling them to adapt to their microenvironment. So far, we have only investigated the impact of organometallic complexes on cytoplasmic or mitochondrial enzymes. Hence, how an alteration of the activity of these enzymes can impact on the ER and the proteostasis process may lead to an ER stress, remains to be understood. Alternatively, the characterization of the impact of metal-based complexes on the activity of redox enzyme present in the ER will certainly be highly informative. A more precise understanding of their impact on the activity of redox enzymes may represent a novel strategy to derive anticancer treatment targeting such enzymes. 

## 9. Perspectives

Over the years, our long-term collaboration allowed Dr. Michel Pfeffer and my group to contribute to a better understanding of the biological properties of Ru and Os organometallic complexes by deciphering part of their mode of action. We were the first academic group to identify the endoplasmic reticulum and the reticulum stress pathway as mediators of their action rather than the induction of DNA damage and the p53 pathway. We demonstrated their localization within the endoplasmic reticulum and identified some of the active mechanisms involved in their uptake. We also identified several redox enzymes whose activity is either affected negatively or positively by the ruthenium complexes and could account for their in vitro and in vivo anticancer properties. Our work also showed that some of these properties are also valid for ruthenium coordinated complexes as well as for complexes with other metals, opening up a new avenue of research.

The potential of the organo-ruthenium/osmium complexes still requires further investigations in multiple directions. For instance, we recently showed that these complexes are potentially useful for photodynamic therapy, based on their property to be light-activated at wavelengths beyond 500 nm to produce reactive oxygen species [52]. Beyond Ruthenium and osmium complexes, other metals can also be used for theranostic approaches using both the light and cytotoxic properties of the metallic complex [53]. Yet, additional work is required to bring these complexes to their ultimate use as anticancer drugs and contribute to the improvement of patient care.

Dr. Michel Pfeffer is now retiring, leaving behind a strong legacy structured around the chemistry and the biological properties of various ruthenium, osmium and other organo-metallic complexes. We do not yet fully perceive all the importance and the implications of his findings, limited as we are by the time and tools at our disposal. Before him, the biological potential of organometallic complexes was largely disregarded, based on the assumption that they do not present the same qualities as the simple coordinated complexes due to the absence of the metal charge conversion within the cells, or based on the assumption that they might be too toxic. Together, we contributed to changing this perception in the field and we were the first researchers to show that ruthenium and osmium complexes are looking past the DNA to be strong inducers of the ER stress pathway. 

I have now collaborated with Dr. Michel Pfeffer for about 15 years. Besides his remarkable scientific contribution, I will greatly miss our discussions and his exemplary attitude as a kind, honest and fair scientist, unwavering on scientific ethics, who should be a model for all of us.
